# Use of Tenofovir Alafenamide Fumarate for HIV Pre-Exposure Prophylaxis and Incidence of Hypertension and Initiation of Statins

**DOI:** 10.1001/jamanetworkopen.2023.32968

**Published:** 2023-09-11

**Authors:** Adovich S. Rivera, Katherine J. Pak, Matthew T. Mefford, Rulin C. Hechter

**Affiliations:** 1 Department of Research and Evaluation, Kaiser Permanente Southern California, Pasadena; 2 Health Systems Science, Kaiser Permanente Bernard J. Tyson School of Medicine, Pasadena, California

## Abstract

**Importance:**

Pre-exposure prophylaxis (PrEP) is an important tool for preventing HIV infection. However, PrEP’s impact on cardiometabolic health is understudied.

**Objective:**

To examine the risk of incident hypertension and statin initiation among adult (age ≥18 years) health plan members starting PrEP with tenofovir alafenamide fumarate (TAF) compared with propensity score–matched adults taking tenofovir disoproxil fumarate (TDF).

**Design, Setting, and Participants:**

This retrospective cohort study used electronic health records (EHRs) from Kaiser Permanente Southern California. Adult members starting PrEP in Kaiser Permanente Southern California between October 2019 and May 2022 were included. Propensity score matching with multiple imputation (50 matched data sets) was conducted to generate 1 TAF:4 TDF matched data sets with balanced baseline covariates.

**Exposures:**

PrEP initiation with either TAF or TDF during the study period.

**Main Outcomes and Measures:**

Incident hypertension and statin initiation within 2 years of PrEP initiation were ascertained through blood pressure and outpatient pharmacy records, respectively. Risk differences and odds ratios (ORs) were estimated using logistic regression and g-computation.

**Results:**

A total of 6824 eligible individuals were identified (mean [SD] age, 33.9 [10.3] years; 6618 [97%] male). This pool was used to generate 2 cohorts without baseline hypertension or statin use for matching (hypertension: n = 5523; statin: n = 6149) In both cohorts, those starting PrEP with TAF were older and were more likely to be non-Hispanic White compared with those starting with TDF. In matched analysis adjusting for baseline covariates, TAF use was associated with elevated risk of incident hypertension (TAF: n = 371; risk difference, 0.81 [95% CI, 0.12-1.50]; OR, 1.64 [95% CI, 1.05-2.56]). TAF use was also associated with elevated risk of statin initiation (TAF: n = 382; risk difference, 0.85 [95% CI, 0.37-1.33]; OR, 2.33 [95% CI, 1.41-3.85]). Subgroup analyses restricted to individuals 40 years and older at PrEP initiation showed similar results with larger risk difference in statin initiation (risk difference, 4.24 [95% CI, 1.82-6.26]; OR, 3.05 [95% CI, 1.64-5.67]).

**Conclusions and Relevance:**

In this study of people taking PrEP, TAF use was found to be associated with higher incident hypertension and statin initiation compared with TDF use, especially in those 40 years or older. Continued monitoring of blood pressure and lipids for TAF users is warranted.

## Key Points

**Question:**

Is initiation of tenofovir alafenamide fumarate (TAF) for pre-exposure prophylaxis (PrEP) for HIV associated with increased risk for incident hypertension and statin initiation compared with tenofovir disoproxil fumarate?

**Findings:**

In this cohort study of PrEP initiators without hypertension at baseline, TAF use was associated with increased hypertension risk. TAF was also associated with statin initiation in a similar analysis limited to PrEP initiators without history of statin use.

**Meaning:**

Closer monitoring of blood pressure and lipid levels may be warranted if using TAF.

## Teaser

This cohort study examines the risk of incident hypertension and statin initiation among adults starting pre-exposure prophylaxis with tenofovir alafenamide fumarate compared with propensity score–matched adults taking tenofovir disoproxil fumarate.

## Introduction

Pre-exposure prophylaxis (PrEP) is an effective intervention for reducing risk of HIV infection and is a key component for HIV elimination efforts in the US.^1,2,3^ As of 2023, 2 daily oral PrEP regimens has been approved by the US Food and Drug Administration: emtricitabine/tenofovir disoproxil fumarate (TDF) was approved in 2012, while emtricitabine/tenofovir alafenamide fumarate (TAF) was approved in 2019.^4,5^ While efficacy trials demonstrated that the 2 regimens are comparable in terms of effects for HIV prevention and overall safety,^6,7,8^ data from the DISCOVER trial^7,8^ showed that individuals taking TAF for PrEP had better bone and kidney health markers than those taking TDF. Further, those in the TAF arm had greater weight gain, while those in the TDF arm experienced greater declines in total cholesterol and low-density lipoprotein cholesterol (LDL-C) during follow-up.^8^ These findings are consistent with studies in people with HIV who demonstrated elevations in weight, total cholesterol, and LDL-C levels associated with TAF use for HIV antiretroviral therapy.^9,10,11,12,13^

Abnormal cholesterol levels (eg, elevated total cholesterol) and greater body weight have been associated with increased cardiovascular disease risk in the general population.^14,15^ In the DISCOVER trial,^16^ despite weight gain in the TAF arm, there was no difference in the initiation of lipid-modifying agents (eg, statins) between the 2 arms; however, risk differences of other cardiometabolic conditions like hypertension were not assessed. Importantly, the generalizability of clinical trial results may be limited due to differences in the selected trial population and the population on PrEP in the real world.^17^

One prior study, which used the TriNetX electronic health records (EHR) research database (n = 9956), assessed if TAF use was associated with a higher risk of cardiometabolic conditions vs TDF use. They reported a higher incidence of statin initiation among those taking TAF compared with propensity score–matched individuals taking TDF. Meanwhile, elevated blood pressure, defined as systolic blood pressure (SBP) more than 140 mm Hg, occurred more often in those taking TDF despite hypertension diagnosis rates being comparable between TDF and TAF users.^18^ These findings need to be confirmed since the use of diagnosis codes or prescriptions separately to identify cardiometabolic conditions may lead to underestimated outcomes.^19^

If trial data are limited, careful analysis of observational studies can be used to compare differences in outcomes among pharmacologic interventions.^20,21^ Here, we compared the risk of incident hypertension and statin initiation among adults initiating PrEP with TAF vs with TDF among members of an integrated health care system in California.

## Methods

### Study Design and Data Source

We conducted a retrospective cohort study of adults initiating PrEP in Kaiser Permanente Southern California (KPSC) using EHRs. KPSC is an integrated health care delivery system providing services to approximately 4.8 million diverse members that is representative of the communities in the Southern California service area.^22,23^ Members’ receipt of health care services are tracked in KPSC’s EHR system. Out-of-system care is captured through billing claims. The KPSC institutional review board approved the study protocol with a waiver of informed consent. Reporting followed Strengthening the Reporting of Observational Studies in Epidemiology (STROBE) reporting guideline.

### Study Population and Follow-Up

We identified adult health plan members (age ≥18 years) initiating PrEP (TAF or TDF) for HIV prevention between October 1, 2019, and May 31, 2022. Individuals with diagnosed HIV infection or chronic kidney disease at any time prior to and up to PrEP initiation were excluded. We further excluded individuals with evidence of abnormal kidney, liver, or hematologic laboratory test results at baseline (eMethods in Supplement 1) To ensure adequate follow-up, those who had fewer than 30 days of membership after their PrEP initiation date were excluded from the analysis.

Two analytic cohorts were derived from the entire study sample: one for assessing the risk of incident hypertension and one for the risk of statin initiation. The 2 cohorts were restricted to those without prevalent hypertension or statin use at baseline, respectively. Each cohort was then used to generate propensity score–matched cohorts (1 TAF:4 TDF) for further analysis.

Individuals in each matched cohort contributed time at risk from the date of PrEP initiation (index date) until censored on the earliest of the following dates: reaching maximum follow-up (2 years), membership disenrollment, death, or end of the observation period (June 30, 2022).

### Main Exposure and Outcomes

The main exposure was PrEP initiation with either TAF or TDF during enrollment at KPSC. Outpatient pharmacy dispensing records were used to identify the first filled PrEP prescription from October 1, 2019, and May 31, 2022. The primary outcomes were incident hypertension and, separately, statin initiation that occurred between 30 days and up to 2 years after PrEP initiation.

Hypertension was ascertained using both *International Statistical Classification of Diseases and Related Health Problems, Tenth Revision* diagnosis codes (I10, I15.xx) and outpatient blood pressure measurements and defined as 2 or more diagnosis codes during separate encounters in any care setting or 2 or more abnormal outpatient blood pressure measurements (SBP ≥140 mm Hg or diastolic blood pressure [DBP] ≥90 mm Hg) occurring on separate dates within a 2-year period. The earliest date between diagnosis codes or abnormal blood pressures was used for the date of hypertension diagnosis. The 140/90 mm Hg cutoff value was used following the Joint National Committee 8 definition and the health care system’s guidelines.^24^ In a sensitivity analysis, a cutoff of SBP/DBP level of 130/80 mm Hg or higher was used following the 2017 American College of Cardiology/American Heart Association hypertension guidelines.^25^ Statin initiation was ascertained using outpatient pharmacy dispensing records for first filled (sold) statin medication during the study period.

### Covariates

Baseline covariates included age, sex, EHR-reported race and ethnicity (Asian [non-Hispanic], Black [non-Hispanic], Hispanic, White [non-Hispanic], and other [eg, Native American/Alaskan, Pacific Islander]), census block group Area Deprivation Index, insurance type (commercial, Medicaid/care, other), cardiometabolic comorbidities (diabetes, dyslipidemia, and/or hypertension), body mass index (calculated as weight in kilograms divided by height in meters squared), estimated glomerular filtration rate (eGFR), lipids (total cholesterol, high-density lipoprotein cholesterol, LDL-C), calculated 10-year atherosclerotic cardiovascular disease (ASCVD) risk score, medical center, and calendar year of the index date.

Diabetes and dyslipidemia were assessed using combinations of diagnosis codes, medication use, and laboratory values (eMethods in Supplement 1).^26,27^ The ASCVD risk score was calculated using the American College of Cardiology/American Heart Association method, which incorporated baseline laboratory, medication, and comorbidities data.^14^ For body mass index, lipids, eGFR, and inputs for the ASCVD risk score, we used the value closest to baseline measured from 12 months before to 7 days after the index date.

### Statistical Analysis

For each analytic cohort, we first calculated summary statistics of baseline covariates to compare all unmatched individuals initiating PrEP with TAF vs with TDF. We then estimated measures of differences in risk (primary: risk difference; secondary: odds ratio [OR], hazard ratio [HR]) using logistic or Cox regression with multiply imputed and matched data.

Multiple imputation with chained equations were first used to impute missing baseline covariates and create 50 imputed data sets (eTable 1 in Supplement 1). In each data set, we performed propensity score matching to address covariate imbalance with each person taking TAF being matched to 4 persons taking TDF (1:4 matching). This ratio was selected to optimize bias-variance trade-off and avoid situations where the TDF group had no events.^28^

The propensity score model for the incident hypertension analysis was a logistic regression models adjusting for baseline age, sex, race and ethnicity, insurance type, medical center, calendar year, clinical measures (body mass index and lipids), ASCVD risk score, cardiometabolic comorbidities (diabetes and dyslipidemia), and Area Deprivation Index. The propensity score models for incident statin initiation analysis adjusted for all covariates listed above plus hypertension.

The 50 matched data sets were then used to estimate risk difference and OR via logistic regression with g-computation. Time-to-event analysis was conducted using Cox proportional hazards regression models to estimate HR. Aside from treatment status, no additional covariates were included in the outcome models.^29^ All models used robust variance estimators to obtain 95% CIs. The results across all multiply imputed and matched data sets were pooled using Rubin’s rules.

Due to matching, we focused on estimates that point to differences in incident outcome risk between TAF users and the same TAF users had they used TDF instead.^29^ We did not account for switching from or discontinuation of initial PrEP type used during follow-up, so estimates are observational analogs of intention-to-treat analyses.

We performed a sensitivity analysis among a subset of individuals 40 years or older at PrEP initiation because practice guidelines recommend statin initiation in this age group and the onset of hypertension is more likely in this age group. Another sensitivity analysis was conducted by defining hypertension using the cutoff value of SBP/DBP levels of 130/80 mm Hg or higher.

All analyses were conducted in R/RStudio version 4.4 (R Foundation) using the *MatchIt*, *MatchThem*, *marginaleffects*, and *mice* packages.^30,31,32,33^ eMethods in Supplement 1 contains full analytical details and sample R code.

## Results

**Figure 1.**
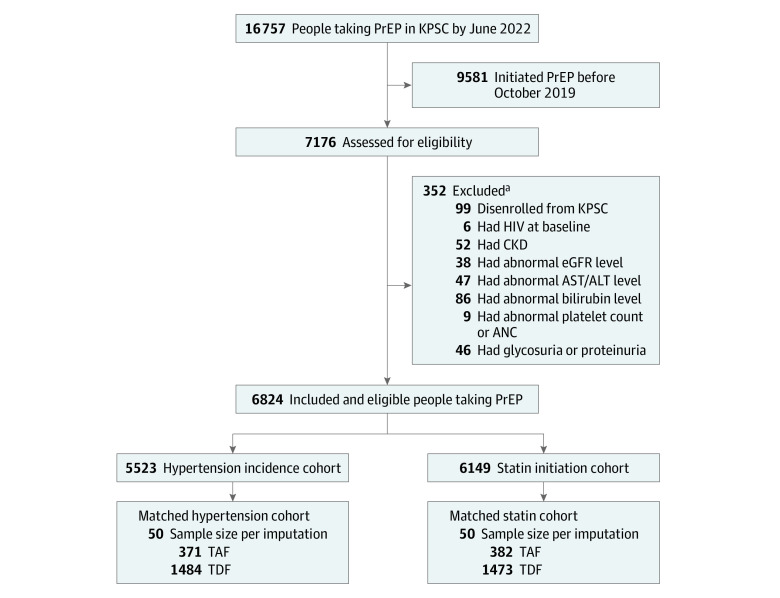
Identification of Eligible Pre-Exposure Prophylaxis (PrEP) Users, Kaiser Permanente Southern California (KPSC), October 2019-June 2022 ANC indicates absolute neutrophil count; AST/ALT, aspartate transaminase/alanine transaminase; CKD, chronic kidney disease; eGFR, estimated glomerular filtration rate; TAF, emtricitabine/tenofovir disoproxil fumarate; TDF, emtricitabine/tenofovir disoproxil fumarate. ^a^Some individuals met multiple exclusion criteria.

There were 6824 eligible individuals starting PrEP included in the main pool for generating the analytic cohorts (Figure 1). The mean (SD) age was 34 (10.3) years and 6618 (97%) were male (eTable 2 in Supplement 1). Compared with those excluded (n = 352), eligible individuals tended to be younger, less likely to have Medicare/Medicaid insurance, more likely taking TDF, and less likely to have baseline cardiometabolic comorbidity.

### Incident Hypertension

**Figure 2.**
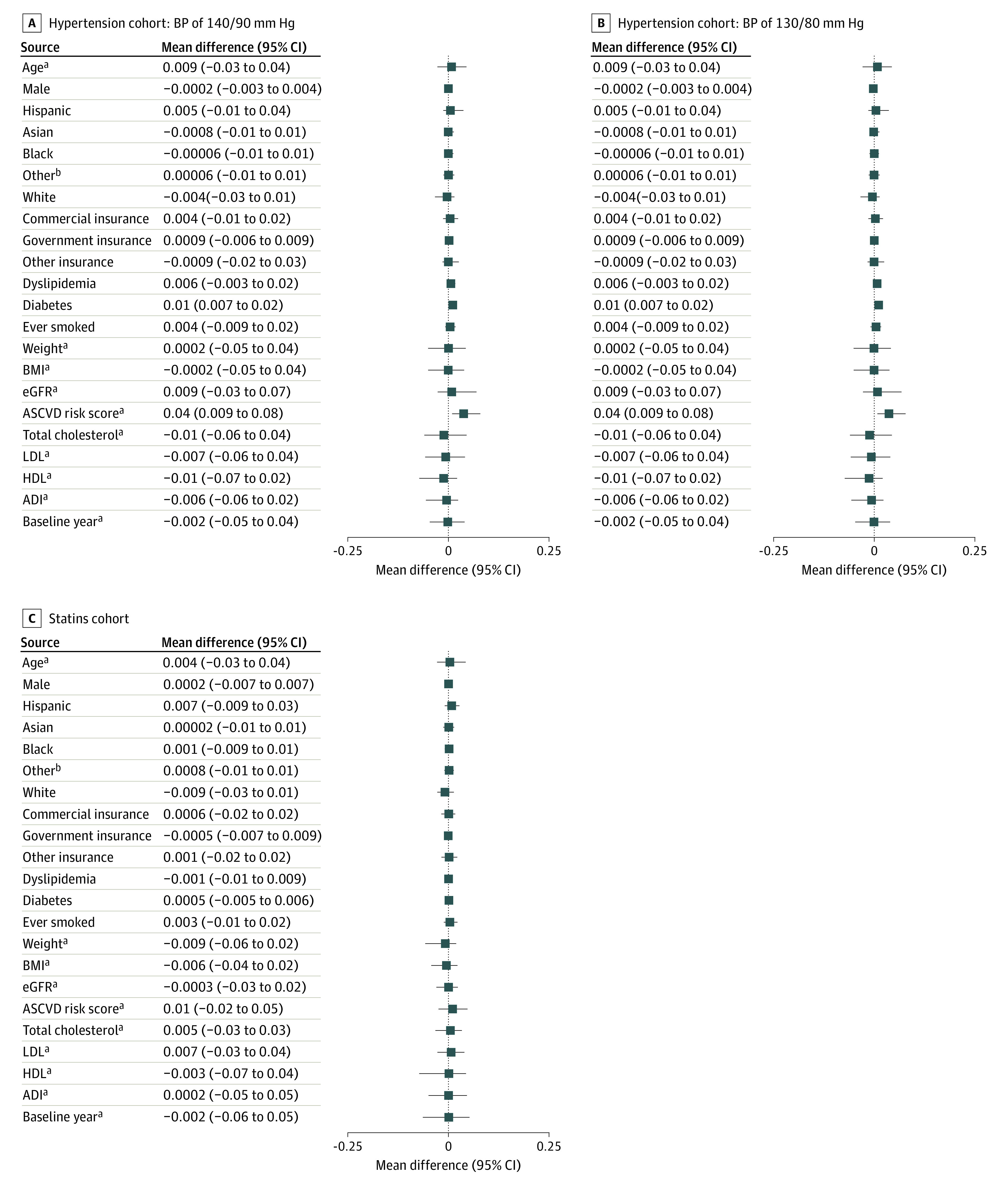
Balance of Baseline Covariates After Matching (50 Matched Data Sets per Cohort) In this figure, −0.1 and 0.1 represent the recommended threshold for assessing balance. Balance in medical centers were also achieved but not reported to protect confidentiality. ADI indicates Area Deprivation Index; ASCVD, atherosclerotic cardiovascular disease; BMI, body mass index; eGFR, estimated glomerular filtration rate; HDL, high-density lipoprotein; LDL, low-density lipoprotein. ^a^Standardized difference is reported instead of raw differences. Raw differences were used to compare proportions of categorical variables. ^b^Other race and ethnicity include Native American/Alaskan, Pacific Islander, multiracial, and all other types of responses not reported in the figure.

We identified a total of 5523 individuals without prevalent (baseline) hypertension (TAF: 371 [6.7%]; TDF: 5152 [93.3%]) prior to matching. Compared with unmatched individuals taking TDF, those taking TAF were older (mean [SD] age, 36 [10.2] vs 33 [9.3] years), more likely to be non-Hispanic White (142 [43%] vs 1609 [34%]), and have diabetes at baseline (16 [4%] vs 73 [1%]) but less likely to be Hispanic (101 [31%] vs 1976 [42%]) and use Medicare/Medicaid (11 [3%] vs 304 [6%]) or commercial (264 [71%] vs 3960 [77%]) insurance. Those taking TAF also had lower eGFR (mean [SD], 102 [18] vs 109 [16] mL/kg/1.73 m^2^), higher ASCVD risk score (mean [SD], 2.6% [4.0%] vs 1.6% [1.8%]), and shorter follow-up (median [IQR], 276 [140-523)] vs 321 [156-561] days) (Table 1). The matched cohort per imputation (50 matched data sets) included 1855 people taking PrEP (TAF: 371 [20%]; TDF: 1484 [80%]). The differences in baseline covariates between those taking TAF vs TDF were reduced after matching and balance in key covariates was achieved (mean differences ≤0.10) (Figure 2A and eFigure A in Supplement 1).

**Table 1.** Baseline Characteristics of Analytic Cohorts, Kaiser Permanente Southern California, October 2019-June 2022

Characteristic	No. (%)					
	Incident hypertension cohort			Statin initiation cohort		
	All (n = 5523)	TAF (n = 371)	TDF (n = 5152)	All (n = 6149)	TAF (n = 382)	TDF (n = 5767)
Age, mean (SD), y	32.8 (9.4)	36.4 (10.2)	32.5 (9.3)	32.9 (9.3)	35.7 (9.6)	32.7 (9.2)
Male	5362 (97.1)	366 (98.7)	4996 (97.0)	5966 (97.0)	376 (98.4)	5590 (96.9)
Female	155 (2.8)	5 (1.3)	150 (2.9)	175 (2.8)	5 (1.3)	170 (2.9)
Race and ethnicity						
Asian, non-Hispanic	550 (11.0)	35 (10.7)	515 (11.0)	602 (10.7)	38 (11.2)	564 (10.7)
Black, non-Hispanic	324 (6.5)	20 (6.1)	304 (6.5)	381 (6.8)	24 (7.1)	357 (6.8)
Hispanic	2077 (41.4)	101 (30.8)	1976 (42.2)	2392 (42.5)	104 (30.6)	2288 (43.3)
White, non-Hispanic	1751 (34.9)	142 (43.3)	1609 (34.3)	1915 (34.0)	143 (42.1)	1772 (33.5)
Other, non-Hispanic^a^	314 (6.3)	30 (9.1)	284 (6.1)	338 (6.0)	31 (9.1)	307 (5.8)
Commercial insurance	4224 (76.5)	264 (71.2)	3960 (76.9)	4719 (76.8)	268 (70.3)	4451 (77.2)
Medicare/Medicaid insurance	315 (5.7)	11 (3.0)	304 (5.9)	369 (6.0)	14 (3.7)	355 (6.2)
Diabetes	89 (1.6)	16 (4.3)	73 (1.4)	99 (1.6)	7 (1.8)	92 (1.6)
Dyslipidemia	255 (4.6)	20 (5.4)	235 (4.6)	NA	NA	NA
Hypertension	NA	NA	NA	799 (13.0)	35 (9.2)	764 (13.2)
Ever smoked	1170 (21.2)	70 (18.9)	1100 (21.4)	1337 (21.7)	76 (19.9)	1261 (21.9)
Block group ADI, mean (SD)	100.0 (18.3)	98.4 (18.5)	100.1 (18.3)	100.3 (18.1)	99.3 (18.0)	100.3 (18.1)
Weight, mean (SD), kg	82.7 (18.6)	81.4 (16.2)	82.8 (18.7)	85.0 (21.0)	82.9 (18.5)	85.1 (21.1)
BMI, mean (SD)	26.7 (5.5)	26.2 (5.1)	26.7 (5.5)	27.4 (6.2)	26.6 (5.6)	27.4 (6.2)
eGFR, mean (SD), mL/min/1.73 m^2^	108.2 (16.1)	101.6 (17.8)	108.5 (16.0)	108.2 (16.1)	102.5 (17.3)	108.5 (16.0)
ASCVD risk, mean (SD)	1.67 (2.00)	2.59 (3.95)	1.62 (1.81)	1.65 (1.71)	2.04 (2.40)	1.62 (1.66)

Abbreviations: ADI, Area Deprivation Index; ASCVD, atherosclerotic cardiovascular disease; BMI, body mass index (calculated as weight in kilograms divided by height in meters squared); eGFR, estimated glomerular filtration rate; NA, not applicable; TAF, tenofovir alafenamide fumarate; TDF, tenofovir disoproxil fumarate.

^a^ Other race and ethnicity include Native American/Alaskan, Pacific Islander, multiracial, and all other types of responses not reported in this table.

Among those taking TAF, 8 (2.2%) developed incident hypertension within 2 years of PrEP initiation. In comparison, 1.3% (95% CI, 1.0%-1.7%) of those taking TDF developed hypertension across the imputations (50 matched data sets). The incident rate for hypertension was 0.06 per 1000 person-years for those taking TAF and 0.04 (95% CI, 0.03-0.05) per 1000 person-years for those in TDF across imputations.

TAF use was associated with a higher likelihood of hypertension within 2 years of PrEP initiation compared with TDF use (risk difference, 0.81% [95% CI, 0.12%-1.5%]; OR, 1.64 [95% CI, 1.05-2.56]). In the time-to-event analysis, the association of TAF use (vs TDF use) with hypertension risk was not statistically significant (HR, 1.63 [95% CI, 0.67-3.96]). The sensitivity analysis limited to those 40 years or older at PrEP initiation showed similar results (Table 2).

**Table 2.** Comparison of Hypertension Risk Between TAF Users and Propensity Score–Matched TDF Users, Kaiser Permanente Southern California, October 2019-June 2022

Outcome	Cumulative incidence per 100 person		Risk difference (95% CI) (null: 0)^b^^,^^c^	Odds ratio (95% CI) (null: 1)^b^^,^^c^	Incidence rate (per 1000 person-years)		Hazard ratio (95% CI) (null: 1)^c^
	TAF users	TDF users (95% CI)^a^			TAF users	TDF users (95% CI)^a^	
**Hypertension (BP, 140/90 mm Hg)**							
All (TAF n = 371)	2.2	1.3 (1.0-1.7)	0.81 (0.12 to 1.5)	1.64 (1.05-2.56)	0.06	0.04 (0.03-0.05)	1.63 (0.67-3.96)
Age ≥40 y at PreP initiation (TAF n = 100)	5	2.6 (2.0-3.2)	2.41 (0.03-4.80)	2.0 (1.07-3.76)	0.15	0.08 (0.04-0.11)	1.97 (0.65-6.00)
**Hypertension (BP, 130/80 mm Hg)**							
All (TAF n = 287)	10.8	5.5 (4.8-6.3)	5.29 (3.44-7.13)	2.08 (1.61-2.70)	0.36	0.18 (0.14-0.22)	2.03 (1.29-3.22)^b^
Age ≥40 y at PreP initiation (TAF n = 69)	17.4	7.7 (6.6-8.8)	9.73 (3.59-15.88)	2.55 (1.50-4.33)	0.56	0.24 (0.16-0.32)	2.32 (1.12-4.81)^b^

Abbreviations: PrEP, pre-exposure prophylaxis; TAF, tenofovir alafenamide fumarate; TDF, tenofovir disoproxil fumarate.

^a^ Matching model covariates include age, sex, race and ethnicity, insurance type, medical center, calendar year, clinical measures (body mass index, lipids), atherosclerotic cardiovascular risk score, cardiometabolic comorbidities (diabetes, dyslipidemia), and Area Deprivation Index. Matching done for each missing data imputation (50 matched data sets). CIs represent Wald-type 95% intervals. No CI for TAF since the same set of TAF users are used across all matching models.

^b^ Confidence interval did not cross the null threshold (0 for risk difference and 1 for odds/hazard ratio). 95% CI does not cross the null.

^c^ Risk difference, odds ratio, and hazard models only adjust for treatment status. Confidence intervals are derived from results for each imputation and pooled using Rubin’s rules.

In a sensitivity analysis using SBP/DBP levels of 130/80 mm Hg or more as the cutoff to define hypertension, we identified 3454 eligible individuals (TAF: 287 [83.1%]; TDF: 3167 [91.7%]) with notable differences in age, race and ethnicity, insurance, smoking status, and eGFR (eTable 3 in Supplement 1). Each imputed matched sample included 1435 individuals (TAF: 287 [20%]; TDF: 1148 [80%]). Matched samples in this sensitivity analysis also exhibited balanced baseline covariates (Figure 2B and eFigure B in Supplement 1). A higher proportion of people taking TAF (31 [10.8%]) were identified with incident hypertension compared with those taking TDF (mean, 5.5%; 95% CI, 4.8%-6.3%; 50 matched data sets). Like the main analysis, we observed that TAF use was associated with a higher risk of hypertension even after accounting for censoring. Similar results were observed in the sensitivity analysis restricted to individuals 40 years or older at baseline (Table 2).

### Statin Initiation

We identified 6149 individuals without history of statin use at baseline to serve as a pool for matching (TAF: 382 [6.2%]; TDF: 5767 [93.8%]). Compared with unmatched individuals taking TDF, those taking TAF were older (mean [SD] age, 36 [9.6] vs 33 [9.2] years), more likely to be non-Hispanic White (143 [42%] vs 1772 [34%]), less likely to be Hispanic (104 [31%] vs 2288 [43%]), less likely to use commercial (268 [70%] vs 4451 [77%]) or Medicare/Medicaid (14 [4%] vs 355 [6%]) insurance, and less likely to have hypertension at baseline (35 [9%] vs 764 [13%]). Those taking TAF tended to have higher ASCVD risk score (mean [SD], 2.0% [2.4%] vs 1.6% [1.7%]) and shorter follow-up duration (median [IQR], 290 [147-538] days vs 324 [157-571] days) (Table 1). Each imputation (50 matched data sets) in the matched cohort included 1855 individuals (TAF: 382 [20.6%]; TDF: 1473 [79.4%]). Covariate balance was achieved after matching (Figure 2C and eFigure C in Supplement 1).

Among individuals taking TAF, 6 (1.6%) initiated statins within 2 years after PrEP initiation. About 1.0% (95% CI, 0.7%-1.3%) of those taking TDF initiated statins across the imputations. The incident rate for statin initiation was 0.05 per 1000 person-years for those taking TAF and 0.03 (95% CI, 0.02-0.04) per 1000 person-years in matched TDF users.

Matched analysis showed a higher likelihood of statin initiation associated with TAF use (risk difference, 0.85% [95% CI, 0.37%-1.33%]; OR, 2.33 [95% CI, 1.41-3.85]) but not with time-to-event analysis (HR, 2.26 [95% CI, 0.76-6.69]). The sensitivity analysis limited to people 40 years or older at PrEP initiation showed larger differences in risk associated with TAF compared with the main analysis (Table 3).

**Table 3.** Comparison of Statin Initiation Risk Between TAF and Matched TDF Users, Kaiser Permanente Southern California, October 2019-June 2022

Outcome	Cumulative incidence per 100 person (%)		Risk difference (95% CI) (null: 0)^b^^,^^c^	Odds ratio (95% CI) (null: 1)^b^^,^^c^	Incidence rate (per 1000 person-years)		Hazard ratio (95% CI) (null: 1)^c^
	TAF	Matched TDF (95% CI)^a^			TAF	Matched TDF (95% CI)^a^	
All (TAF n = 382)	1.6	1.0 (0.7-1.3)	0.85 (0.37-1.33)	2.33 (1.41-3.85)	0.05	0.03 (0.02-0.04)	2.26 (0.76-6.69)
Age ≥40 y at PrEP initiation (TAF n = 92)	6.5	3.6 (2.6-4.6)	4.24 (1.82-6.66)	3.05 (1.64-5.67)	0.18	0.10 (0.06-0.15)	2.72 (0.87-8.45)

Abbreviations: PrEP, pre-exposure prophylaxis; TAF, tenofovir alafenamide fumarate; TDF, tenofovir disoproxil fumarate.

^a^ Matching covariates include age, sex, race and ethnicity, insurance type, medical center, calendar year, clinical measures (body mass index, lipids), atherosclerotic cardiovascular risk score, cardiometabolic comorbidities (diabetes, dyslipidemia, hypertension), and Area Deprivation Index. Matching done for each missing data imputation (50 matched data sets). CIs represent Wald-type 95% intervals. No CI for TAF since the same set of TAF users are used across all matching models.

^b^ Confidence interval did not cross the null threshold (0 for risk difference and 1 for odds/hazard ratio). 95% CI does not cross the null.

^c^ Risk difference, odds ratio, and hazard models only adjust for treatment status. Confidence intervals are derived from results for each imputation and pooled using Rubin’s rules.

## Discussion

We found that TAF use was associated with higher risk of incident hypertension and statin initiation compared with propensity score–matched TDF users within 2 years after PrEP initiation using logistic regression. Time-to-event analyses suggested no differences except for the sensitivity analysis for hypertension using the cutoff of 130/80 mm Hg or higher. Our finding on increased statin initiation risk contrasts with DISCOVER but aligns with the TriNetX study.^16,18^ However, our findings on increased hypertension risk associated with TAF contrasts with the TriNetX study, which showed no difference in risk. Importantly, among adults 40 years or older, we found that the risk difference for statin initiation associated with TAF vs TDF use was greater than the overall cohort, suggesting the possibility of age-specific differences in risk due to TAF use.

The higher occurrence of hypertension and statin initiation may be due to weight and lipid-level changes associated with TAF as observed in DISCOVER and in studies of people with HIV.^5,8,34^ In people with HIV, those who initiated antiretroviral therapy containing TAF exhibited greater weight gain and increased LDL-C compared with those undergoing antiretroviral therapy containing TDF.^13^ Similarly, switching from TDF to TAF in people with HIV was associated with significant increase in weight and worsening of lipid profiles.^10,11^ The weight gain may affect blood pressure and hypertension risk through neurohormonal pathways controlling sodium balance.^35^ Meanwhile, statins are generally initiated when clinicians observe changes in lipid profiles that affect cardiovascular risk. Despite observed metabolic changes, DISCOVER did not report differences in initiation of lipid-modifying agents and argued that metabolic changes (eg, greater weight gain in TAF arm, steeper LDL changes in TDF arm) were minimal and not likely clinically meaningful.^8^ Similarly, a switching study in people with HIV did not find differences in incident cardiometabolic events.^36^ Finally, since we used TDF as a comparator, our findings could also be due to potential decrease in cardiometabolic risk from TDF. The TDF arm in the iPrEx trial had lower weight gain and short-term LDL-C decline compared with placebo.^37^ Future mediation work assessing the role of PrEP-associated metabolic changes on cardiometabolic risk would be important.

### Limitations

Confounding by indication (eg, those at higher risk of hypertension were more likely to use TAF) is a main limitation of observational studies. To mitigate this, we used matching to identify TDF users that have similar propensity to initiate with TAF based on observed baseline covariates. Importantly, matching requires proper specification of the matching model and inclusion of all important confounders.^29^ However, structured data from EHRs may not capture all relevant covariates, the observed elevated risk in TAF users may be partially due to residual confounding.

Other limitations should be considered when interpreting our findings. First, the health care system’s first dispensing record of PrEP was used to identify initiation, which may lead to misclassification if a person had started PrEP before their KPSC enrollment. Second, our results are analogs of intention-to-treat analyses. They do not account for adherence or discontinuation and cannot be used to compare risks according to cumulative exposures or early switching. Third, since TAF was relatively low in our sample, those taking TAF might represent early adopters who are likely different from those taking TDF. We included comorbidities and neighborhood conditions in our propensity score model, but we cannot account for factors not recorded in the EHR. Fourth, we had a limited follow-up time and low number of events. We set a 2-year maximum follow-up based on TAF’s approval year and cohort attrition. However, this limited our ability to study long-term risks. Relatedly, the limited events could explain nonsignificant main time-to-event results. Our estimated HRs represent the mean of HRs during the entire follow-up period and not period-specific HRs.^38^ Null HR may fail to identify treatment effect in the presence of nonproportional hazards or significantly different period-specific HRs (eg, presence of immediate effects but no delayed effects). Due to low events, we were unable to use methods that accounts for nonproportional hazards in our analysis to estimate period-specific HRs.^39^ Fifth, our sample included primarily male individuals; thus, we were unable to investigate sex-related effect modification. Relatedly, we did not investigate if receipt of gender-affirming hormone therapy modifies the association of TAF with cardiometabolic conditions. Sixth, there was high missingness of the baseline ASCVD risk score from missing lipids data. This was addressed through multiple imputation.^40^ Relatedly, low events combined with high missingness of follow-up weight (approximately 70%) and lipids (approximately 90%), precluded mediation analysis through marginal structural models.^41,42^ Finally, our study was conducted in a single integrated health care system in the US with unique demographics so our findings may also be less generalizable to individuals in other health care settings such as those serving primarily uninsured individuals or outside the US.

## Conclusions

We found an elevated risk of hypertension and statin initiation among TAF users especially among those who initiated PrEP at age 40 years and older. TAF has been a welcome addition to the products for PrEP due to its benefits on kidney and bone health and smaller pill size.^43^ However, it may have unwanted impact on cardiometabolic health. Future studies with larger sample size and longer follow-up period are warranted to provide more evidence to inform clinical decision-making regarding different PrEP regimens, especially among those with increased risk for cardiometabolic disease.

## Notes

Supplement 1. **eMethods.** Additional Details on Definitions, Imputation, Matching and Modeling **eTable 1.** Missingness of Covariates Used in Imputation Models **eTable 2.** Comparison of Eligible and Excluded PreP Users, Kaiser Permanente Southern California, October 2019 – June 2022 **eTable 3.** Analytic Cohort Using Alternative Hypertension Definition, Kaiser Permanente Southern California, October 2019 – June 2022 **eFigure.** Balance of Baseline Covariates Before Matching (m=50 per cohort)

Supplement 2.
Data Sharing Statement

## References

[1] Fauci AS, Redfield RR, Sigounas G, Weahkee MD, Giroir BP. Ending the HIV epidemic: a plan for the United States. JAMA. 2019;321(9):844-845. doi:10.1001/jama.2019.1343 30730529

[2] Owens DK, Davidson KW, Krist AH, ; US Preventive Services Task Force. Preexposure prophylaxis for the prevention of HIV infection: US Preventive Services Task Force recommendation statement. JAMA. 2019;321(22):2203-2213. doi:10.1001/jama.2019.6390 31184747

[3] Global AIDS strategy 2021-2026. UNAIDS. Accessed August 8, 2023. https://www.unaids.org/sites/default/files/media_asset/global-AIDS-strategy-2021-2026_en.pdf

[4] Kirby T. Cabotegravir, a new option for PrEP. Lancet Infect Dis. 2020;20(7):781. doi:10.1016/S1473-3099(20)30497-7 32592669

[5] Wassner C, Bradley N, Lee Y. A review and clinical understanding of tenofovir: tenofovir disoproxil fumarate versus tenofovir alafenamide. J Int Assoc Provid AIDS Care. Published online April 15, 2020. doi:10.1177/2325958220919231 32295453 PMC7163232

[6] Pilkington V, Hill A, Hughes S, Nwokolo N, Pozniak A. How safe is TDF/FTC as PrEP? A systematic review and meta-analysis of the risk of adverse events in 13 randomised trials of PrEP. J Virus Erad. 2018;4(4):215-224. doi:10.1016/S2055-6640(20)30312-5 30515300 PMC6248833

[7] Mayer KH, Molina J-M, Thompson MA, . Emtricitabine and tenofovir alafenamide vs emtricitabine and tenofovir disoproxil fumarate for HIV pre-exposure prophylaxis (DISCOVER): primary results from a randomised, double-blind, multicentre, active-controlled, phase 3, non-inferiority trial. Lancet. 2020;396(10246):239-254. doi:10.1016/S0140-6736(20)31065-5 32711800 PMC9665936

[8] Ogbuagu O, Ruane PJ, Podzamczer D, ; DISCOVER study team. Long-term safety and efficacy of emtricitabine and tenofovir alafenamide vs emtricitabine and tenofovir disoproxil fumarate for HIV-1 pre-exposure prophylaxis: week 96 results from a randomised, double-blind, placebo-controlled, phase 3 trial. Lancet HIV. 2021;8(7):e397-e407. doi:10.1016/S2352-3018(21)00071-0 34197772 PMC12492902

[9] Cid-Silva P, Fernández-Bargiela N, Margusino-Framiñán L, . Treatment with tenofovir alafenamide fumarate worsens the lipid profile of HIV-infected patients versus treatment with tenofovir disoproxil fumarate, each coformulated with elvitegravir, cobicistat, and emtricitabine. Basic Clin Pharmacol Toxicol. 2019;124(4):479-490. doi:10.1111/bcpt.13161 30388308

[10] Sun L, He Y, Xu L, . Higher risk of dyslipidemia with coformulated elvitegravir, cobicistat, emtricitabine, and tenofovir alafenamide than efavirenz, lamivudine, and tenofovir disoproxil fumarate among antiretroviral-naive people living with HIV in China. J Acquir Immune Defic Syndr. 2022;91(S1):S8-S15. doi:10.1097/QAI.0000000000003040 36094509

[11] Kauppinen KJ, Kivelä P, Sutinen J. Switching from tenofovir disoproxil fumarate to tenofovir alafenamide significantly worsens the lipid profile in a real-world setting. AIDS Patient Care STDS. 2019;33(12):500-506. doi:10.1089/apc.2019.0236 31742421

[12] Mallon PW, Brunet L, Hsu RK, . Weight gain before and after switch from TDF to TAF in a U.S. cohort study. J Int AIDS Soc. 2021;24(4):e25702. doi:10.1002/jia2.25702 33838004 PMC8035674

[13] Venter WDF, Moorhouse M, Sokhela S, . Dolutegravir plus two different prodrugs of tenofovir to treat HIV. N Engl J Med. 2019;381(9):803-815. doi:10.1056/NEJMoa1902824 31339677

[14] Goff DC Jr, Lloyd-Jones DM, Bennett G, ; American College of Cardiology/American Heart Association Task Force on Practice Guidelines. 2013 ACC/AHA guideline on the assessment of cardiovascular risk: a report of the American College of Cardiology/American Heart Association Task Force on Practice Guidelines. Circulation. 2014;129(25)(suppl 2):S49-S73. doi:10.1161/01.cir.0000437741.48606.98 24222018

[15] Lloyd-Jones DM, Allen NB, Anderson CAM, ; American Heart Association. Life’s essential 8: updating and enhancing the American Heart Association’s construct of cardiovascular health: a presidential advisory from the American Heart Association. Circulation. 2022;146(5):e18-e43. doi:10.1161/CIR.0000000000001078 35766027 PMC10503546

[16] Mounzer K, Clarke A, Doblecki-Lewis S, . Lipid parameters and lipid-modifying agent use in participants initiating F/TAF or F/TDF for PrEP in the DISCOVER Trial. Presented at: 24th International AIDS Conference; 2022; Montreal, Canada. Accessed May 18, 2023. https://www.natap.org/2022/IAC/IAC_17.htm

[17] Hojilla JC, Hurley LB, Marcus JL, . Characterization of HIV preexposure prophylaxis use behaviors and HIV incidence among US adults in an integrated health care system. JAMA Netw Open. 2021;4(8):e2122692. doi:10.1001/jamanetworkopen.2021.22692 34436609 PMC8391097

[18] Yap AG. Metabolic adverse effects of different HIV pre-exposure prophylaxis medications in minority populations: a United States multicenter research network propensity-matched analysis. University of Texas at Austin; 2022. Accessed August 8, 2023. https://repositories.lib.utexas.edu/handle/2152/117151

[19] Banerjee D, Chung S, Wong EC, Wang EJ, Stafford RS, Palaniappan LP. Underdiagnosis of hypertension using electronic health records. Am J Hypertens. 2012;25(1):97-102. doi:10.1038/ajh.2011.179 22031453 PMC3600431

[20] Wang SV, Schneeweiss S, Franklin JM, ; RCT-DUPLICATE Initiative. Emulation of randomized clinical trials with nonrandomized database analyses: results of 32 clinical trials. JAMA. 2023;329(16):1376-1385. doi:10.1001/jama.2023.4221 37097356 PMC10130954

[21] Hernán MA, Sauer BC, Hernández-Díaz S, Platt R, Shrier I. Specifying a target trial prevents immortal time bias and other self-inflicted injuries in observational analyses. J Clin Epidemiol. 2016;79:70-75. doi:10.1016/j.jclinepi.2016.04.014 27237061 PMC5124536

[22] Koebnick C, Langer-Gould AM, Gould MK, . Sociodemographic characteristics of members of a large, integrated health care system: comparison with US Census Bureau data. Perm J. 2012;16(3):37-41. doi:10.7812/TPP/12-031 23012597 PMC3442759

[23] Davis AC, Voelkel JL, Remmers CL, Adams JL, McGlynn EA. Comparing Kaiser Permanente members to the general population: implications for generalizability of research. Perm J. 2023;27(2):87-98. doi:10.7812/TPP/22.172 37170584 PMC10266863

[24] Armstrong C; Joint National Committee. JNC8 guidelines for the management of hypertension in adults. Am Fam Physician. 2014;90(7):503-504. 25369633

[25] Whelton PK, Carey RM, Mancia G, Kreutz R, Bundy JD, Williams B. Harmonization of the American College of Cardiology/American Heart Association and European Society of Cardiology/European Society of Hypertension blood pressure/hypertension guidelines: comparisons, reflections, and recommendations. Circulation. 2022;146(11):868-877. doi:10.1161/CIRCULATIONAHA.121.054602 35950927

[26] Oake J, Aref-Eshghi E, Godwin M, . Using electronic medical record to identify patients with dyslipidemia in primary care settings: international classification of disease code matters from one region to a national database. Biomed Inform Insights. Published online February 10, 2017. doi:10.1177/1178222616685880 28469428 PMC5391192

[27] Rivera AS, Rusie L, Plank M, . Association of cumulative viral load with the incidence of hypertension and diabetes in people With HIV. Hypertension. 2022;79(11):e135-e142. doi:10.1161/HYPERTENSIONAHA.122.19302 36378919 PMC9673163

[28] Austin PC. Statistical criteria for selecting the optimal number of untreated subjects matched to each treated subject when using many-to-one matching on the propensity score. Am J Epidemiol. 2010;172(9):1092-1097. doi:10.1093/aje/kwq224 20802241 PMC2962254

[29] Greifer N, Stuart EA. Matching methods for confounder adjustment: an addition to the epidemiologist’s toolbox. Epidemiol Rev. 2022;43(1):118-129. doi:10.1093/epirev/mxab003 34109972 PMC9005055

[30] van Buuren S, Groothuis-Oudshoorn K. mice: Multivariate imputation by chained equations in R. J Stat Softw. 2011;45(3):1-67. doi:10.18637/jss.v045.i03

[31] Ho D, Imai K, King G, Stuart EA. MatchIt: nonparametric preprocessing for parametric causal inference. J Stat Softw. 2011;42(8). doi:10.18637/jss.v042.i08

[32] Pishgar F, Greifer N, Leyrat C, Stuart E. MatchThem: matching and weighting after multiple imputation. arXiv. Preprint posted online September 24, 2020. doi:10.48550/arXiv.2009.11772

[33] Arel-Bundock V. marginaleffects 0.13.0. Accessed August 8, 2023. https://vincentarelbundock.github.io/marginaleffects/

[34] Fields SD, Tung E. Patient-focused selection of PrEP medication for individuals at risk of HIV: a narrative review. Infect Dis Ther. 2021;10(1):165-186. doi:10.1007/s40121-020-00384-5 33569743 PMC7875561

[35] Hall JE, do Carmo JM, da Silva AA, Wang Z, Hall ME. Obesity-induced hypertension: interaction of neurohumoral and renal mechanisms. Circ Res. 2015;116(6):991-1006. doi:10.1161/CIRCRESAHA.116.305697 25767285 PMC4363087

[36] Martínez-Sanz J, Serrano-Villar S, Muriel A, . Metabolic-related outcomes after switching from tenofovir disoproxil fumarate to tenofovir alafenamide in adults with human immunodeficiency virus (HIV): a multicenter prospective cohort study. Clin Infect Dis. 2023;76(3):e652-e660. doi:10.1093/cid/ciac621 35903910

[37] Glidden DV, Mulligan K, McMahan V, . Metabolic effects of preexposure prophylaxis with coformulated tenofovir disoproxil fumarate and emtricitabine. Clin Infect Dis. 2018;67(3):411-419. doi:10.1093/cid/ciy083 29415175 PMC6051460

[38] Stensrud MJ, Hernán MA. Why test for proportional hazards? JAMA. 2020;323(14):1401-1402. doi:10.1001/jama.2020.1267 32167523 PMC11983487

[39] Hernán MA. The hazards of hazard ratios. Epidemiology. 2010;21(1):13-15. doi:10.1097/EDE.0b013e3181c1ea43 20010207 PMC3653612

[40] Lee JH, Huber JCJ Jr. Evaluation of multiple imputation with large proportions of missing data: how much is too much? Iran J Public Health. 2021;50(7):1372-1380. doi:10.18502/ijph.v50i7.6626 34568175 PMC8426774

[41] Vourli G, Touloumi G. Performance of the marginal structural models under various scenarios of incomplete marker’s values: a simulation study. Biom J. 2015;57(2):254-270. doi:10.1002/bimj.201300159 25352223

[42] VanderWeele TJ. Invited commentary: structural equation models and epidemiologic analysis. Am J Epidemiol. 2012;176(7):608-612. doi:10.1093/aje/kws213 22956513 PMC3530375

[43] D’Angelo AB, Westmoreland DA, Carneiro PB, Johnson J, Grov C. Why Are patients switching from tenofovir disoproxil fumarate/emtricitabine (Truvada) to tenofovir alafenamide/emtricitabine (Descovy) for pre-exposure prophylaxis? AIDS Patient Care STDS. 2021;35(8):327-334. doi:10.1089/apc.2021.0033 34375141 PMC8380788

