# Supplementary material for: Use of Tenofovir Alafenamide Fumarate for HIV Pre-Exposure Prophylaxis and Incidence of Hypertension and Initiation of Statins
Source: JAMA Netw Open. 2023 Sep 11;6(9):e2332968. doi: 10.1001/jamanetworkopen.2023.32968 (PMC10495863; doi:10.1001/jamanetworkopen.2023.32968)
Supplement: Supplement 1. — eMethods. Additional Details on Definitions, Imputation, Matching and Modeling eTable 1. Missingness of Covariates Used in Imputation Models eTable 2. Comparison of Eligible and Excluded PreP Users, Kaiser Permanente Southern California, October 2019 – June 2022 eTable 3. Analytic Cohort Using Alternative Hypertension Definition, Kaiser Permanente Southern California, October 2019 – June 2022 eFigure. Balance of Baseline Covariates Before Matching (m=50 per cohort) [file jamanetwopen-e2332968-s001.pdf]

Supplemental Online Content

Rivera AS, Pak KJ, Mefford MT, Hechter RC. Use of tenofovir alafenamide fumarate for HIV pre-exposure prophylaxis and incidence of hypertension and initiation of statins. *JAMA Netw Open*. 2023;6(9):e2332968. doi:10.1001/jamanetworkopen.2023.32968

eMethods. Additional details on Definitions, Imputation, Matching and Modeling

eTable 1. Missingness of Covariates used in Imputation models

eTable 2. Comparison of Eligible and Excluded PreP users, Kaiser Permanente Southern California, October 2019 – June 2022

eTable 3. Analytic Cohort using Alternative Hypertension Definition, Kaiser Permanente Southern California, October 2019 – June 2022

eFigure. Balance of Baseline Covariates Before Matching (m=50 per cohort)

This supplemental material has been provided by the authors to give readers additional information about their work.

© 2023 Rivera AS et al. *JAMA Network Open.*

eMethods. Additional details on Definitions, Imputation, Matching and Modeling

A. Operational definitions for selected covariates

Dyslipidemia is defined as having At least 2 ICD codes (ICD9: 272, ICD10: E78.0, E78.00, E78.01, E78.1, E78.2, E78.3, E78.4, E78.41, E78.49, E78.5) for dyslipidemia within 2-year period or abnormal Lipid levels plus initiation of statin within a 6-month period.

Diabetes is defined as having (i) any one ICD code (ICD9: 250.x, ICD10: E10.x, E11.X, E13.X) AND (HbA1c >= 6.5% OR use of anti-diabetes medication) OR (ii) having at least two ICD codes for diabetes

Census block group is calculated using the *sociome* R package.

Atherosclerotic cardiovascular disease risk was the 10-year risk score calculated using the *CVrisk* R package. Risk were still calculated for people less than 40 years old although in practice the formulas are usually just used for people 40 or older.

Estimated glomerular filtration rate was calculated using the CKD-EPI Creatinine equation 2021 which does not include race in the formula.

B. Eligibility criteria for labs

To be eligible for analysis, individuals should not have evidence of abnormal laboratory results at baseline. Abnormal labs are:

- Estimated Glomerular filtration rate < 60 mL/min/1.73m^2
- Alanine transaminase ≥ 2.5 upper limit of normal (ULN) (ULN = 63)
- Aspartate transaminase ≥ 2.5 ULN (ULN = 34)
- Total bilirubin > 1.5 mg/dL
- Direct bilirubin < 0.3 mg/dL
- Absolute neutrophil count < 1000/mm^3
- Platelets < 75/mm^3
- Hemoglobin <10 g/dL
- Grade 3 or 4 glucosuria
- Grade 3 or 4 proteinuria

C. Sequence of analytical steps

The first step in the analysis was to address missing baseline covariates using multiple imputation with chained equations (m=50). In the second step, we addressed imbalance between TAF and TDF users through 1:4 propensity score matching separately for each imputed dataset. Propensity scores were estimated using a multivariable logistic regression model with the following covariates: age, sex, race & ethnicity, insurance type, medical center, calendar year,

© 2023 Rivera AS et al. *JAMA Network Open.*

clinical measures (BMI, lipids), ASCVD risk score, cardiometabolic comorbidities, and ADI. For the incident hypertension analysis, comorbidities included diabetes and dyslipidemia. Meanwhile, the statin initiation analysis included diabetes, dyslipidemia, and hypertension. Balance after matching was assessed using love plots where we treat balance as achieved if absolute mean differences were less than or equal to 0.10.

In the third step, we used the imputed and matched cohort data to estimate RD and OR via logistic regression with g-computation. We also estimated hazard ratios (HR) using Cox proportional hazards regression models to account for different follow-up times. Aside from treatment status, no additional covariates were included in the outcome models. All models used robust variance estimators to obtain 95% confidence intervals (CI). Finally, the results from modeling each imputed and matched data were pooled using Rubin's rules.

D. Missing data imputation

Missing covariate data were imputed using multiple imputation with chained equations as implemented in the *mice* package. The missing data model included the following baseline covariates: age, gender, race and ethnicity, insurance, ASCVD risk score, eGFR, weight, LDL-C, HDL-C, TC, cardiometabolic comorbidities, and community variables. Community variables at the census tract level included racial composition, median household income, proportion with high school education or higher, income disparity, and area deprivation index. We also included the Nelson-Aalen estimator for the corresponding outcome. We ran separate imputation models for each analytic cohort/outcome and generated 50 imputations for each run.

E. Propensity Score Matching model

© 2023 Rivera AS et al. *JAMA Network Open.*

Propensity score matching was based on a generalized logistic regression model and implemented using the *MatchIt* and *MatchThem* packages. During exploratory analyses, we tried (1) using covariate balancing propensity scores instead of the logistic model, (2) optimal instead of nearest neighbor, and (3) full instead of nearest neighbor matching. We found that none of these achieved better balance than the default generalized logistic model. Balance was assessed by examining love plots and summary statistics.

Matching was chosen over weighting due to the poor performance of weighting during initial analyses. Importantly, this choice affected the estimand. Matching does not allow inference about the full analytical cohort, instead we are only able to estimate counterfactual scenarios for the TAF-treated individuals. That is we can answer the question: “What is the risk of the outcome among the people in the data who initiated TAF had they initiated with TDF instead?” This corresponds to an observational analog of the “average treatment effect among the treated” rather than the more commonly reported estimate of “average treatment effect”.

F. Estimating risk difference, odds ratio, and hazard ratio

To obtain risk differences and odds ratios, we first ran a logistic regression model using the matched data and adjusting only for treatment status. We then use the comparisons command of the *marginaleffects* package with appropriate transformations to calculate the risk difference. Due to matching, the target population for these estimates were those who initiated PrEP with TAF.

Hazard ratios were estimated using a Cox proportional hazards model with the survival package. No covariates aside from treatment status were included in the model.

© 2023 Rivera AS et al. *JAMA Network Open.*

Since we created 50 imputed datasets using multiple imputation, we repeated the matching and outcome estimation for each imputation. For all models, we specified the pair ids as the clustering variable to calculate robust standard errors and confidence intervals. The results were then pooled following Rubin's rules.

d. Sample R code (with statin initiation as the outcome)

```
####Libraries
library(tidyr)
library(dplyr)
library(janitor)
library(lubridate)
library(mice)
library(MatchThem)
library(cobalt)
library(parallel)
library(fastDummies)
library(marginaleffects)
library(survival)

####Multiple imputation

#a. Load and clean wide data
o1_new_fin <- readRDS("prep – o1 – o1_new_fin – 28 feb 2023.RDS")
mod.data <- o1_new_fin %>%
  filter(!is.na(prep_type1)) %>%
  filter(included==1) %>%
  filter(!is.na(enroll_start_dt)) %>%
  filter(index_dt <= study_end_date) %>%
  #at least 30 days of follow-up
  filter(Time_event_statin >= 30) %>%
  filter(statin_bl==0) %>%
  mutate(A = ifelse(prep_type1=='TAF',1,0)) %>%
  mutate(Y_1 = statin_inc)

#b. Set-up multiple imputation data
mi.data <- mod.data %>% ungroup() %>%
  filter(statin_bl == 0) %>%
  dplyr::rename(event_dt = statin_event_dt,
                Time_event_2yrs = Time_event_statin_2yrs) %>%
  mutate(Y_1 = statin_inc) %>%
  dplyr::select(mrn, fips_bg, geoyr,
```

© 2023 Rivera AS et al. *JAMA Network Open.*

```
                Y_1, A, statin_gap,
                age_bl, male_gender, race_5cat,
                ins_comm, ins_gov, ins_oth,
                htn_bl, dm_bl, ever_smoked_bl,
                tc_0, hdl_0, ldl_0, cvrisk_0,
                ra_as, ra_bl, ra_wh, ra_his, adi_ct, adi_bg,
                medcen_bl, wt_kg_0, bmi_0, gfr_bl, index_yr,
                gap_enr_prep_days,
                index_dt, enroll_end_dt, statins_start_dt,
                ltfu_dt, event_dt, prep_end_dt1, Time_event_2yrs)

#c. Add Area deprivation index indicators
temp <- adi_bg_indicators %>%
  dplyr::select(geoyr, fips_bg, h_med_income,
                education3_7, income_disparity)

mi.data <- left_join(mi.data, temp, by=c('geoyr','fips_bg')) %>%
  distinct(mrn, .keep_all=TRUE) %>%
  dplyr::select(-fips_bg, -geoyr) %>%
  mutate(income_disparity = ifelse(is.infinite(income_disparity),NA,income_disparity)) %>%
  ungroup()

#d. Calculate nelson-Aalen estimator
mi.data$na_est <- nelsonaalen(mi.data, timevar='Time_event_2yrs', statusvar='Y_1')

#e. Center continuous variables
mi.data <- mi.data %>% ungroup() %>%
  mutate(age_bl_orig = age_bl) %>%
  mutate(age_bl = age_bl - mean(mi.data$age_bl, na.rm=TRUE),
         cvrisk_0 = cvrisk_0 - mean(mi.data$cvrisk_0, na.rm=TRUE),
         gfr_bl = gfr_bl - mean(mi.data$gfr_bl, na.rm=TRUE),
         wt_kg_0 = wt_kg_0 - mean(mi.data$wt_kg_0, na.rm=TRUE),
         ldl_0 = ldl_0 - mean(mi.data$ldl_0, na.rm=TRUE),
         hdl_0 = hdl_0 - mean(mi.data$hdl_0, na.rm=TRUE),
         tc_0 = tc_0 - mean(mi.data$tc_0, na.rm=TRUE),
         ra_as = ra_as - mean(mi.data$ra_as, na.rm=TRUE),
         ra_bl = ra_bl - mean(mi.data$ra_bl, na.rm=TRUE),
         ra_wh = ra_wh - mean(mi.data$ra_wh, na.rm=TRUE),
         ra_his = ra_his - mean(mi.data$ra_his, na.rm=TRUE),
         adi_ct = adi_ct - mean(mi.data$adi_ct, na.rm=TRUE),
         adi_bg = adi_bg - mean(mi.data$adi_bg, na.rm=TRUE),
         index_yr = index_yr - mean(mi.data$index_yr, na.rm=TRUE),
         h_med_income = h_med_income - mean(mi.data$h_med_income, na.rm=TRUE),
         education3_7 = education3_7 - mean(mi.data$education3_7, na.rm=TRUE),
         income_disparity = income_disparity - mean(mi.data$income_disparity, na.rm=TRUE))
```

© 2023 Rivera AS et al. *JAMA Network Open.*

```
#f. Set-up imputation parameters and controls
temp <- mice::mice(mi.data, m = 1, maxit=0, seed=12345)
meth <- temp$method
meth[["statins_start_dt"]] <- ""
pred <- temp$pred
vars_exclude_mice <- c('mrn','age_bl_orig', 'statin_gap', 'Time_event_2yrs',
                    'index_dt', 'enroll_end_dt', 'statins_start_dt',
                     'ltfu_dt', 'event_dt', 'prep_end_dt1')
pred[,vars_exclude_mice] <- 0
pred[vars_exclude_mice,] <- 0

#g. Generate and save imputed datasets (m=50)
imputed.datasets <- mice::mice(mi.data, m = 50, maxit=5,
                              method=meth, predictorMatrix=pred,
                              seed=12345)

saveRDS(imputed.datasets, 'prep - o1 - imputed datasets - statins.RDS')

####Matching

#a. Load imputed datasets
imputed.datasets <- readRDS('prep - o1 - imputed datasets - statins.RDS')

#b. Extract datasets into long format
temp <- complete(imputed.datasets, 'long', include=TRUE)
imputed.datasets <- as.mids(temp)

##c. Set-up  matching model formula
wt.form <- formula('A ~ age_bl + male_gender + race_5cat +
                  ins_comm + ins_gov + ins_oth +
                  htn_bl + dm_bl + ever_smoked_bl +
                  wt_kg_0 + bmi_0 + gfr_bl + cvrisk_0 +
                  tc_0 + ldl_0 + hdl_0 +
                  medcen_bl + adi_bg + index_yr')

#d. Generate and save matches with imputed datasets
matched.datasets.glm <- matchthem(wt.form,
                                imputed.datasets ,
                                approach = 'within',
                                estimand='ATT',
                                method = 'nearest',
                                distance='glm',
                                ratio=4)
saveRDS(matched.datasets.glm, 'prep - o1 - m - glm - statins - all.RDS')

#e. Examine love plot
```

© 2023 Rivera AS et al. *JAMA Network Open.*

```
v <- data.frame(old = c('medcen_bl_11', 'medcen_bl_06','medcen_bl_07',
                   'medcen_bl_01','medcen_bl_02','medcen_bl_04','medcen_bl_05',
                    'medcen_bl_09','medcen_bl_03','medcen_bl_18','medcen_bl_19',
                   'medcen_bl_10','medcen_bl_17','medcen_bl_13','medcen_bl_21',
                   'medcen_bl_20','medcen_bl_30','medcen_bl_12','medcen_bl_14',
                   'age_bl', 'male_gender',
                    'race_5cat_hispanic','race_5cat_black', 'race_5cat_white',
                    'race_5cat_asian','race_5cat_other',
                      'ins_comm', 'ins_gov', 'ins_oth',
                      'htn_bl', 'dm_bl', 'ever_smoked_bl',
                      'wt_kg_0', 'bmi_0', 'gfr_bl', 'cvrisk_0',
                      'tc_0', 'ldl_0', 'hdl_0',
                    'adi_bg', 'index_yr'),
             new = c(
               'Med center 11', 'Med center 06','Med center 07',
               'Med center 01','Med center 02','Med center 04','Med center 05',
               'Med center 09','Med center 03','Med center 18','Med center 19',
               'Med center 10','Med center 17','Med center 13','Med center 21',
               'Med center 20','Med center 30','Med center 12','Med center 14',
               'Age', 'Male gender',
               'R&E: Hispanic','R&E: Black', 'R&E: White',
               'R&E: Asian','R&E: Other',
               'Insurance: Commercial', 'Insurance: Government', 'Insurance: Other',
               'Hypertension', 'Diabetes', 'Ever Smoked',
               'Weight', 'BMI', 'GFR', 'ASCVD risk score',
               'Total Cholesterol', 'LDL', 'HDL',
               'ADI', 'Baseline Year'))

lp.statins <- love.plot(matched.datasets.glm.all.statin,
        stats = c("m"),
        colors=c('#E1BE6A','#40B0A6'),
        shapes=c(15,19),
        thresholds = c(m = .1),
        stars='std',
        sample.names=c("Unmatched", "Matched"),
        var.names=v,
        title="Statins cohort",
        drop.distance = TRUE) +
        scale_x_continuous(breaks=c(-0.4,-0.2,-0.1,0,0.1,0.2,0.4))
lp.statins

####Calculating pooled risk difference and odds ratio

#a. Define function to get Risk difference and odds ratio
get_matched_rd_or_results <- function(md){
```

© 2023 Rivera AS et al. *JAMA Network Open.*

```
  require(marginaleffects)
  fits <- lapply(complete(md, "all"), function(d){
    glm(Y_1 ~ A,
        weights=weights,
        family=quasibinomial(),
        data = d)
  })
  comp.imp.rd <- lapply(fits, function(fit) {
    comparisons(fit,
                variables = "A",
                vcov = ~subclass,
                newdata = subset(fit$data, A == 1),
                wts = "weights",
                transform_pre='difference' #default
    )
  })
  comp.imp.or <- lapply(fits, function(fit) {
    comparisons(fit,
                variables = "A",
                vcov = ~subclass,
                newdata = subset(fit$data, A == 1),
                wts = "weights",
                transform_pre = "lnoravg")
  })
  df <- fits[[1]]$df.residual

  or.res <- tidy(mice::pool(comp.imp.or, dfcom=df), conf.int = TRUE, exp=TRUE)
#fits[[1]]$df.residual
  rd.res <- tidy(mice::pool(comp.imp.rd, dfcom=df), conf.int = TRUE) #fits[[1]]$df.residual

  res <- rbind(or.res %>% mutate(output='or'),
               rd.res %>% mutate(output='rd')) %>%
    dplyr::rename(theta=estimate, theta.ll=conf.low, theta.ul=conf.high)
  return(res)
}

#b. Calculate
get_matched_rd_or_results(matched.datasets.glm.all.statin)

#### Calculating pooled hazard ratio

#a. Define function to get pooled hazard ratio
get_pooled_survival <- function(matched.data){
  require(survival)
  #from Noah: add an event marker
  #Time_event2=censor at 2years, Y_1 = event up to 2years
```

```
  x <- with(matched.data,
            coxph(Surv(Time_event_2yrs, Y_1) ~ A, robust = TRUE,
                  weights = weights, cluster = subclass))
  res <- as.data.frame(summary(pool(x))) %>%
    clean_names() %>%
    mutate(theta=exp(estimate),
           theta.ll=exp(estimate-1.96*std_error),
           theta.ul=exp(estimate+1.96*std_error))
  return(list(model=x, res=res))
}

#b. Calculate pooled hazard ratio
get_pooled_survival(matched.datasets.glm.all.statin)
```

© 2023 Rivera AS et al. *JAMA Network Open.*

eTable 1. Missingness of Covariates used in Imputation models.

Covariate[1]	Percent with Missing Data		
	Hypertension (140/90 mmHg)	Hypertension (130/80 mmHg)	Statin initiation
	n = 5523	n = 3454	n = 6149
ASCVD risk score	61.8%	64.0%	61.3%
LDL[2]	47.7%	48.9%	47.7%
HDL[2]	45.9%	46.9%	45.8%
Total Cholesterol[2]	44.2%	45.4%	44.2%
ADI block group	38.1%	39.8%	37.9%
ADI census tract[2]	36.9%	38.9%	36.7%
BMI	23.7%	30.1%	22.5%
Income disparity (census tract) [2]	22.7%	24.4%	22.6%
Median household income (census tract) [2]	22.3%	24.1%	22.2%
Proportion with at least high school education (census tract) [2]	22.3%	24.1%	22.2%
Weight in kilogram	22.1%	28.4%	20.9%
eGFR	11.4%	13.3%	10.9%
Race and ethnicity	9.2%	11.6%	8.5%
Medical center	2.5%	1.9%	2.6%
Insurance, commercial	0.1%	0.0%	0.1%
Insurance, Medicaid or Medicare	0.1%	0.0%	0.1%

Note: **[1] -** Variables that were part of the imputation model but not reported in the table had zero missingness. [2] - Variables included in imputation model but not in the matching or outcome models. Abbreviations: ADI – area deprivation index based on census block group, ASCVD – atherosclerotic cardiovascular disease, BMI – body mass index, eGFR – estimated glomerular filtration rate, HDL – high-density lipoprotein, LDL – low-density lipoprotein.

© 2023 Rivera AS et al. *JAMA Network Open.*

eTable 2. Comparison of Eligible and Excluded PreP users, Kaiser Permanente Southern California, October 2019 – June 2022

	Excluded (n=352)	Included (n=6824)
Age, mean (SD), years	37.4 (13.5)	33.9 (10.3)
Male gender, n (%)	338 (96.0)	6618 (97.0)
Race and Ethnicity, n (%)		
Asian, non-Hispanic	30 (9.2)	659 (10.5)
Black, non-Hispanic	23 (7.1)	424 (6.8)
Hispanic	125 (38.3)	2615 (41.8)
White, non-Hispanic	132 (40.5)	2186 (35.0)
Other, non-Hispanic*	16 (4.9)	369 (5.9)
Insured – Commercial, n (%)	188 (74.3)	5204 (76.3)
Insured – Medicare/Medicaid, n (%)	33 (13.0)	445 (6.5)
Diabetes at baseline, n (%)	67 (19.0)	238 (3.5)
Dyslipidemia at baseline, n (%)	13 (3.7)	304 (4.5)
Hypertension at baseline, n (%)	87 (24.7)	1050 (15.4)
Ever smoked at baseline, n (%)	91 (25.9)	1539 (22.6)
On TDF, n (%)	316 (89.8)	6383 (93.5)
Duration of Follow-up, median (IQR)±, days	386 (190, 631)	401 (199, 679)

Note: * - Other race and ethnicity include multiracial, Native American/Alaskan, Pacific Islander, and all other types of responses not reported in the table. ± - Duration of follow-up is

© 2023 Rivera AS et al. *JAMA Network Open.*

based on date of first record PrEP to the earlier between end of enrollment or study end date (June 30, 2022). Abbreviations: ADI – area deprivation index based on census block group, ASCVD – atherosclerotic cardiovascular disease, BMI – body mass index, eGFR – estimated glomerular filtration rate, IQR – interquartile range, PrEP – pre-exposure prophylaxis, SD – standard deviation, TAF – tenofovir alafenamide fumarate, TDF – tenofovir disoproxil fumarate

© 2023 Rivera AS et al. *JAMA Network Open.*

eTable 3. Analytic Cohort using Alternative Hypertension Definition, Kaiser Permanente Southern California, October 2019 – June 2022.

	**All (n=3,454)**	**TAF (n=287)**	**TDF (n=3,167)**
Age, mean (SD), years	32.4 (8.9)	35.9 (9.8)	32.0 (8.7)
Male gender, n (%)	3360 (97.3)	284 (99.0)	3076 (97.1)
Race and Ethnicity, n (%)			
Asian, non-Hispanic	364 (11.9)	29 (11.7)	335 (11.9)
Black, non-Hispanic	188 (6.2)	12 (4.8)	176 (6.3)
Hispanic	1158 (37.9)	74 (29.8)	1084 (38.7)
White, non-Hispanic	1109 (36.3)	109 (44.0)	1000 (35.7)
Other, non-Hispanic*	233 (7.6)	24 (9.7)	209 (7.5)
Insured - Commercial, n (%)	2630 (76.2)	204 (71.1)	2426 (76.6)
Insured – Medicare/Medicaid, n (%)	137 (4.0)	6 (2.1)	131 (4.1)
Diabetes at baseline, n (%)	35 (1.0)	7 (2.4)	28 (0.9)
Dyslipidemia at baseline, n (%)	73 (2.1)	9 (3.1)	64 (2.0)
Hypertension at baseline, n (%)	-	-	-
Ever smoked at baseline, n (%)	660 (19.1)	45 (15.7)	615 (19.4)
block group ADI, mean (SD)	99.5 (18.2)	97.45 (18.13)	99.63 (18.23)
Weight, mean (SD), kg	80.2 (17.6)	79.2 (14.9)	80.2 (17.7)
BMI, mean (SD), kg/m^2	25.9 (5.1)	25.4 (4.6)	25.9 (5.1)

© 2023 Rivera AS et al. *JAMA Network Open.*

eGFR, mean (SD), mL/min/1.73m^2	107.9 (16.1)	101.8 (17.8)	108.2 (15.9)
ASCVD risk, mean (SD)	1.49 (1.78)	2.22 (4.05)	1.43 (1.47)
Follow-up duration, median (IQR)±, days	280 (139, 484)	230 (106, 466)	283 (139, 485)

Notes: * - Other race and ethnicity include multiracial, Native American/Alaskan, Pacific Islander, and all other types of responses not reported in the table. ± - Duration of follow-up is based on date of first record PrEP to the earlier between end of enrollment or study end date (June 30, 2022). ADI – area deprivation index based on census block group, ASCVD – atherosclerotic cardiovascular disease, BMI – body mass index, eGFR – estimated glomerular filtration rate, IQR – interquartile range, PrEP – pre-exposure prophylaxis, SD – standard deviation, TAF – tenofovir alafenamide fumarate, TDF – tenofovir disoproxil fumarate

© 2023 Rivera AS et al. *JAMA Network Open.*

eFigure. Balance of Baseline Covariates Before Matching (m=50 per cohort)

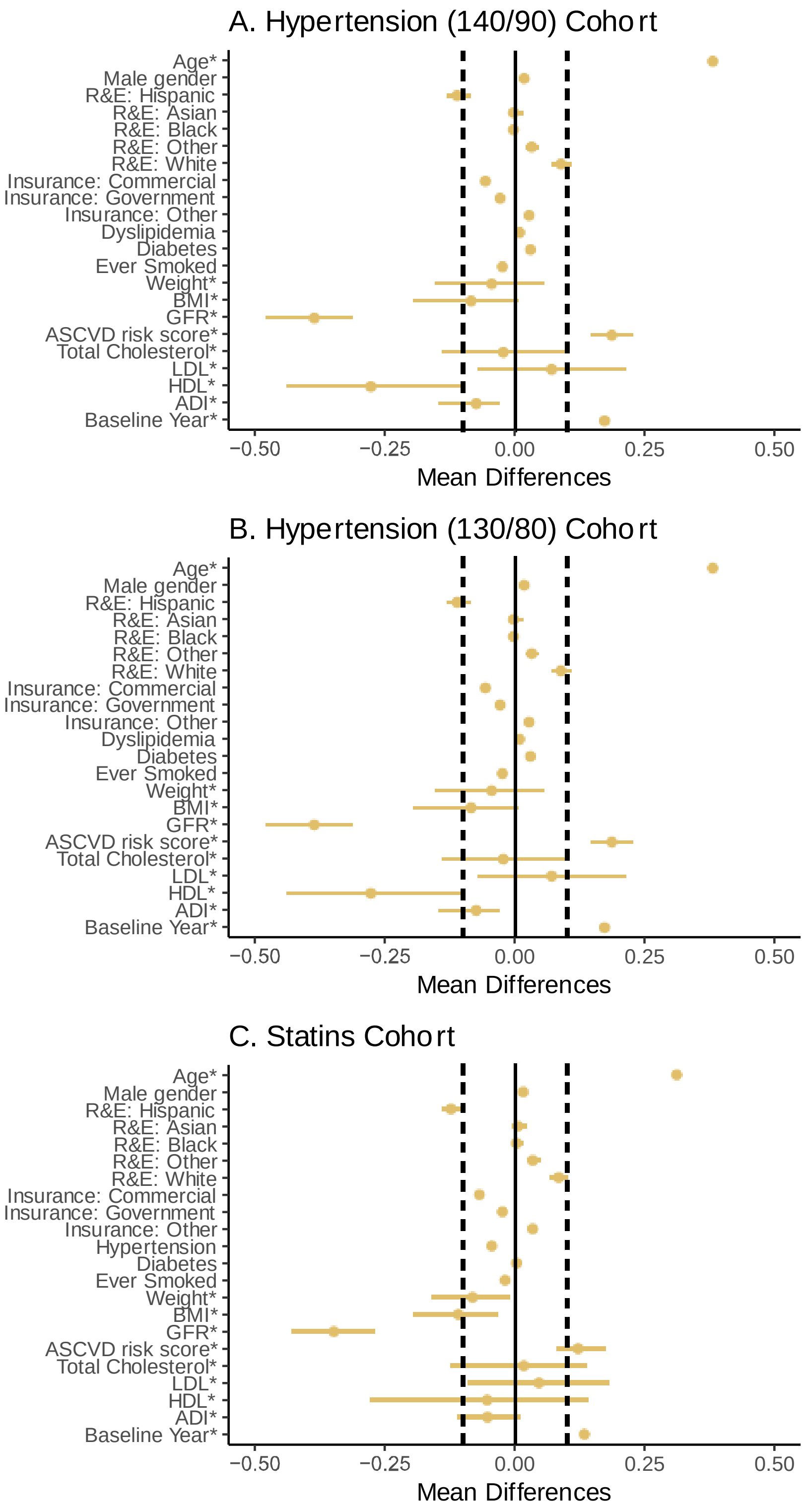

© 2023 Rivera AS et al. *JAMA Network Open.*

Note: * - denotes standardized difference instead of raw differences. Raw differences were used to compare proportions of categorical variables. Horizontal lines show the range of balance measures across imputations. Dashed vertical lines at -0.1 and 0.1 represent the recommended threshold for assessing balance. Balance is achieved if mean differences lie between the two dashed lines. Abbreviations: ADI – area deprivation index, ASCVD – atherosclerotic cardiovascular disease, BMI – body mass index, GFR – estimated glomerular filtration rate, HDL – high density lipoprotein, LDL – low-density lipoprotein, R&E – race and ethnicity. Other race and ethnicity include multiracial, Native American/Alaskan, Pacific Islander, and all other types of responses not reported in the figure.

© 2023 Rivera AS et al. *JAMA Network Open.*
