# Supplementary material for: Use of Tenofovir Alafenamide Fumarate for HIV Pre-Exposure Prophylaxis and Incidence of Hypertension and Initiation of Statins
Source: JAMA Netw Open. 2023 Sep 11;6(9):e2332968. doi: 10.1001/jamanetworkopen.2023.32968 (PMC10495863; doi:10.1001/jamanetworkopen.2023.32968)
Supplement: Supplement 2. — Data Sharing Statement [file jamanetwopen-e2332968-s002.pdf]

Data Sharing Statement

Rivera. Use of Tenofovir Alafenamide Fumarate for HIV Pre-Exposure Prophylaxis and Incidence of Hypertension and Initiation of Statins. *JAMA Netw Open*. Published September 08, 2023. doi:10.1001/jamanetworkopen.2023.32968

Data
Data available: No

Additional Information
Explanation for why data not available: Analysis used data from electronic health records which cannot be easily shared without safeguards on data privacy and confidentiality. Anonymized data that support the findings of this study may be made available from the investigative team in the following conditions: 1) agreement to collaborate with the study team on all publications, 2) provision of external funding for administrative and investigator time necessary for this collaboration, 3) demonstration that the external investigative team is qualified and has documented evidence of training for human subjects protections, and 4) agreement to abide by the terms outlined in data use agreements between institutions
